# Supplementary material for: Reviving product states in the disordered Heisenberg chain
Source: Nat Commun. 2023 Sep 20;14:5847. doi: 10.1038/s41467-023-41464-7 (PMC10511451; doi:10.1038/s41467-023-41464-7)
Supplement: Supplementary file 1 — Supplementary Information [file 41467_2023_41464_MOESM1_ESM.pdf]

Reviving product states in the disordered Heisenberg chain - Supplementary Information

Henrik Wilming, Tobias J. Osborne, Kevin S.C. Decker, and Christoph Karrasch

Supplementary Note 1: Fidelities

In Supplementary Figure 1, we show the fidelities $1-\epsilon = \min_\pm |\langle\Psi(0)_\pm|\Phi(0)_\pm\rangle|^2$ associated with the data of Fig. 2 (main text). One can see that the superposition of two energy eigenstates $|\Psi(0)_\pm\rangle$ is in general very well approximated by a product state $|\Phi(0)_\pm\rangle$. While a small fidelity necessarily yields a small certified amplitude, a large fidelity alone is not sufficient to obtain a large value of $A_{\text{cert.}}$ (right panel).

Supplementary Note 2: Single-particle excitations

For completeness, we briefly discuss a second class of oscillating product states that exist in the disordered Heisenberg chain. In contrast to the deformed domain wall states, these states can be classified as "single-particle like" as they exist irrespective of the strength of the interaction term $\hat{S}^{(z)}_j\hat{S}^{(z)}_{j+1}$ in the Hamiltonian and since their total magnetization is close to minimal. Yet, their energies are extensive. For an explicit construction, consider the single-particle subspace spanned by the states that result from flipping a single spin in the vacuum state $|\downarrow\rangle\otimes\cdots\otimes|\downarrow\rangle$. The Hamiltonian H acts on these states exactly as a single-particle Hamiltonian with nearest-neighbor hoppings plus an additional random potential. It then follows from the theory of Anderson localization [84] that the eigenstates $|\epsilon : k\rangle$ within this subspace are exponentially localized around the different lattice sites $k = 1 \ldots L$ with a localization length that decreases with the strength of the disorder. All these states have a total magnetization $-L/2 + 1/2$ and a large overlap with the product state for which spin k is flipped as compared to the vacuum. Then the states

$$|\psi_k\rangle = \frac{1}{\sqrt{2}}\left(|\downarrow\rangle\otimes\cdots\otimes|\downarrow\rangle + |\epsilon : k\rangle\right) \tag{1}$$

can be approximated to high fidelity by a product state and show high fidelity oscillations at site k for sufficiently high disorder strength.

Supplementary Note 3: The MBL crossover in terms of certified amplitudes

The data in the main part of the paper was obtained for a disorder strength $W = 8$. We now study the behavior of the certified amplitude as one crosses into the ergodic regime. In this case, the eigenstates can no longer be represented faithfully by MPS with a low bond dimension, which entails that we can only access small systems in our numerics.

In Supplementary Figure 2, we show the certified amplitude for $L = 20$ as a function of W. The data was obtained using a maximum bond dimension of $\chi = 48$ (note that even for $L = 20$ this bond dimension is not sufficient to faithfully represent all states for small values of W). We observe that A_{cert} drops to a small value around $W \sim 3.5$, which is the commonly-found disorder strength for the crossover into the ergodic regime at $L = 20$ [33]. This is plausible since our construction hinges on the existence of localized states.

Supplementary Note 4: Multiple localized dynamical excitations

We argued that in a sufficiently large system there will be product states with multiple oscillating, localized excitations at different locations of the chain. In order to back up this claim, we start out with an analytic argument. Let us consider $|E : k_1\rangle$ as well as an eigenstate $|\tilde{E} : k_2\rangle$ obtained from an up-down domain-wall seed at position $k_2 > k_1$ (i.e., $|\text{dw} : k_2\rangle$ but with all spins flipped). Let m be the site at the midpoint between k_1 and k_2 (or the closest site to the right), and let L and R be the set of sites left and right of m, respectively (R includes m). If the distance $d = k_2 - k_1$ is sufficiently large, then both eigenstates $|E : k_1\rangle$ and $|\tilde{E} : k_2\rangle$ are (approximately) unentangled over the bipartition L:R:

$$|E : k_1\rangle \approx |E : k_1\rangle_{\text{L}} \otimes |E : k_1\rangle_{\text{R}}, \tag{2}$$

$$|\tilde{E} : k_2\rangle \approx |\tilde{E} : k_2\rangle_{\text{L}} \otimes |\tilde{E} : k_2\rangle_{\text{R}}. \tag{3}$$

Since $|E, k_1\rangle$ and $|E, k_1 + 1\rangle$ coincide far away from k_1, we also find

$$\left|\Psi^{(k_1)}_\pm\right\rangle = \frac{1}{\sqrt{2}}\left(|E : k_1\rangle \pm |E : k_1 + 1\rangle\right) \tag{4}$$

$$\approx \frac{1}{\sqrt{2}}\left(|E : k_1\rangle_{\text{L}} \pm |E : k_1 + 1\rangle_L\right) \otimes |E : k_1\rangle_{\text{R}} \tag{5}$$

$$= \left|\Psi^{(k_1)}_\pm\right\rangle_{\text{L}} \otimes \left|\Psi^{(k_1)}_\pm\right\rangle_{\text{R}}, \tag{6}$$

and similarly

$$\left|\tilde{\Psi}^{(k_2)}_\pm\right\rangle = \frac{1}{\sqrt{2}}\left(|\tilde{E} : k_2\rangle \pm |\tilde{E} : k_2 + 1\rangle\right) \tag{7}$$

$$\approx \left|\tilde{\Psi}^{(k_2)}_\pm\right\rangle_{\text{L}} \otimes \left|\tilde{\Psi}^{(k_2)}_\pm\right\rangle_{\text{R}}. \tag{8}$$

Now let us consider the state

$$\left|\Psi^{(k_1,k_2)}_{\pm\pm}\right\rangle = \left|\Psi^{(k_1)}_\pm\right\rangle_{\text{L}} \otimes \left|\tilde{\Psi}^{(k_2)}_\pm\right\rangle_{\text{R}} \tag{9}$$

as well as

$$|E : k_1, k_2\rangle = |E : k_1\rangle_{\text{L}} \otimes |\tilde{E} : k_2\rangle_{\text{R}}. \tag{10}$$

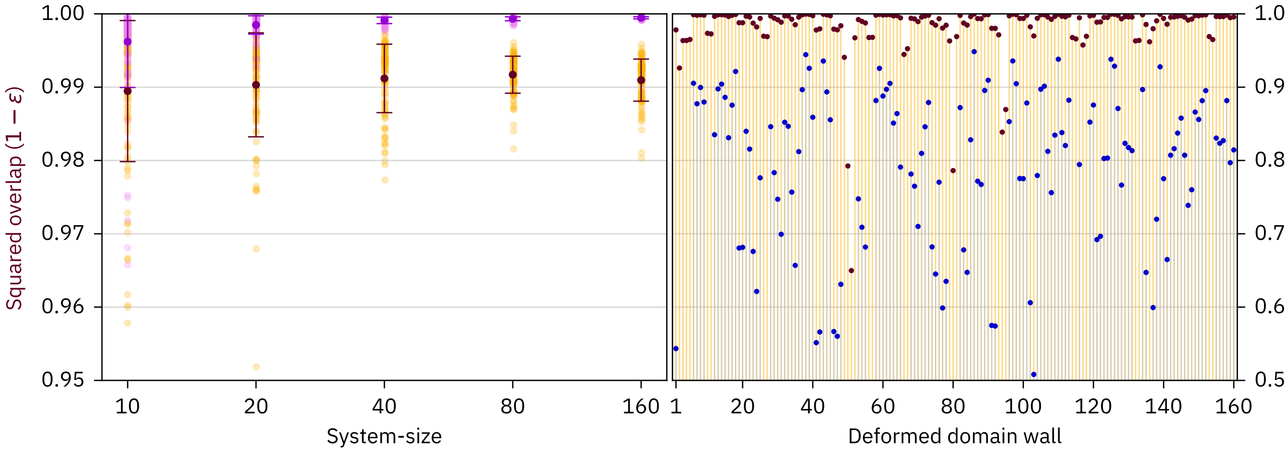

SUPPL. FIG. 1. **Fidelities.** Same as Fig. 2 (main text) but showing the fidelities $1 - \epsilon = \min_{\pm} |\langle \Psi(0)_{\pm} | \Phi(0)_{\pm} \rangle|^2$ of the superposition of eigenstates and the corresponding product state. *Left:* Median (orange) and maximum (light violet) of the fidelities as well as the corresponding mean and standard deviation (brown, dark violet). The maximum is again restricted to domain walls with interface in the middle half of the system. The standard deviation is plotted according to the scale on the left axis. For example, for $L = 10$ it is of the order of 0.01 for the median fidelities and 0.005 for the maximum fidelities. *Right:* Fidelities (brown) and certified amplitudes (blue).

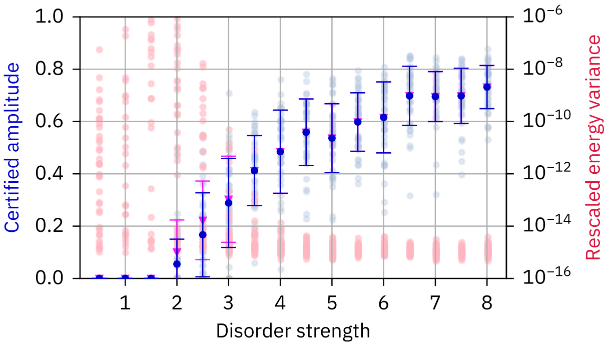

SUPPL. FIG. 2. **Crossover into the ergodic regime.** The same as in Fig. 2 (main text) but as a function of the disorder strength W for a fixed system size $L = 20$ and 40 disorder realizations per disorder strength. The light blue dots show the median certified amplitude for each realization, and dark blue indicates the corresponding average values and the standard deviation. While some of the rescaled energy variances (light red) are no longer small as one crosses into the ergodic regime around $W \sim 3.5$, the picture does not change significantly when post-selecting only on those disorder realizations with median rescaled energy variances below 10^{-12} (magenta).

In case that $|E : k_1, k_2\rangle$, $|E : k_1+1, k_2\rangle$, $|E : k_1, k_2+1\rangle$, and $|E : k_1+1, k_2+1\rangle$ are all eigenstates of $\hat{H}$, then $|\Psi^{(k_1,k_2)}_{\pm\pm}\rangle$ will show perfect revivals. Since $|\Psi^{(k_1)}_{\pm}\rangle$ and $|\tilde{\Psi}^{(k_2)}_{\pm}\rangle$ are well approximated by product states and host a local, oscillating observable, the same holds true for $|\Psi^{(k_1,k_2)}_{\pm\pm}\rangle$.

In order to see whether the system hosts pairs of localized oscillations, it is thus sufficient to show that the states $|E : k_1, k_2\rangle$ are indeed energy eigenstates for sufficiently large d. This can be done efficiently since the states $|E : k_1\rangle$ and $|\tilde{E} : k_2\rangle$ are already available in MPS form. Indeed, if $A^{[j]\sigma_j}$ and $B^{[j]\sigma_j}$ denotes corresponding tensors, then we can simply construct a candidate MPS for $|E : k_1, k_2\rangle$ with local tensors $C^{[j]\sigma_j}$ by setting

$$C^{[j]\sigma_j} = \begin{cases} A^{[j]\sigma_j} & \text{if } j < m \\ B^{[j]\sigma_j} & \text{if } j \geq m. \end{cases} \tag{11}$$

Since the original MPS are (to high precision) product states over the cut at site m, the resulting MPS reproduces the expectation values in the state $|E : k_1\rangle$ in L and those of $|\tilde{E} : k_2\rangle$ in R. In Suppl. Fig. 3 (left), we have calculated the energy variance of this MPS as a function of the distance d for one disorder realization and arbitrary values of $k_1 < k_2$ (with $d = k_2 - k_1$ fixed). The data shows that the resulting state is an eigenstate up to numerical precision for $d \gtrapprox 15 - 20$. (Note that in contrast to the remaining data in this work, we here had to compute both the down-up and the up-down deformed domain-wall eigenstates.)

This distance (or rather the inverse of the exponential decay-rate in Suppl. Fig. 3 (left)) may be interpreted as (twice) the localization length of the system. We emphasize, however, that this localization length will typically fluctuate with the position in the system depending on the precise disorder realization.

From our discussion, it should be clear that the above arguments can be iterated in order to construct eigenstates with three or more domain-wall interfaces by cutting and gluing MPSs. In Suppl. Fig. 3 (right), we plot rescaled energy variances for three domain wall interfaces. Again, we find energy eigenstates up to numerical precision for sufficiently large distances between the interfaces.

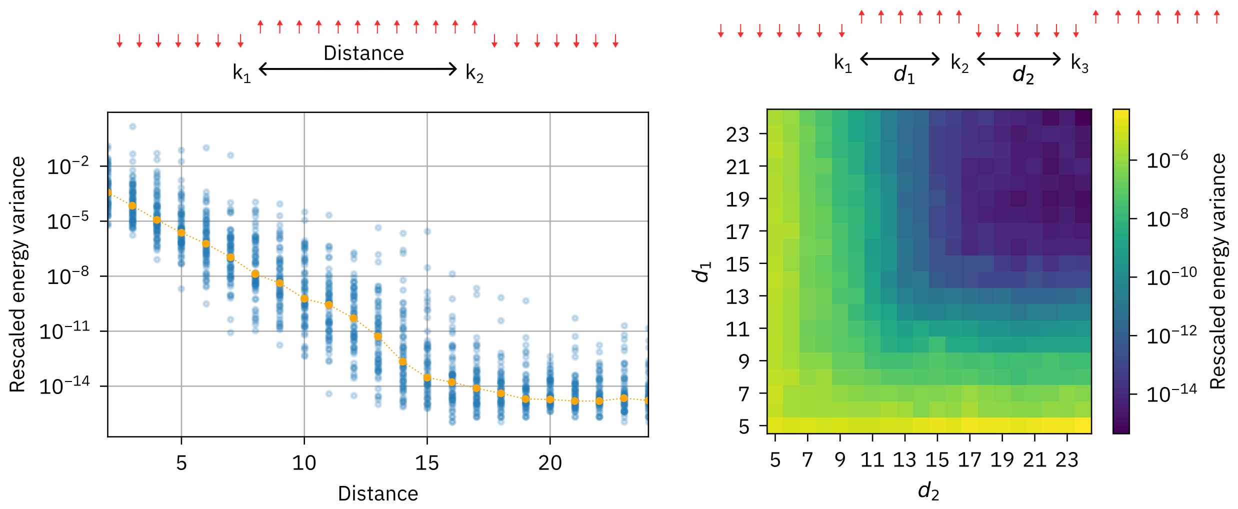

SUPPL. FIG. 3. **Numerical data in support of multiple excitations.** *Left:* For a system of size $L = 80$ and one disorder realization at $W = 8$, we determine all eigenstates associated with down-up domain walls at site k_1 and up-down domain walls at site k_2. For a given distance $d = k_2 - k_1$ between their interfaces, we then cut and glue their respective MPS representations at the midpoint to obtain a candidate state with a pair of localized excitations. Blue points show the associated rescaled energy variance (for varying $k_1 < k_2$ but fixed d), the orange points are the corresponding median. For sufficiently large d, we obtain energy eigenstates. *Right:* The same but for triplets of localized excitations separated by $d_1 = k_2 - k_1 > 0$ and $d_2 = k_3 - k_2 > 0$. We vary $30 \le k_2 \le 49$ and show the corresponding median.

Supplementary Note 5: Approximate eigenstates and timescales

Our numerical procedure yields matrix-product states $|\psi\rangle$ that are approximate eigenstates in the sense that their energy variance $\mathrm{Var}(|\psi\rangle, \hat{H}) = \langle\psi|\hat{H}^2|\psi\rangle - \langle\psi|\hat{H}|\psi\rangle^2$ is on the order of machine precision. This entails that all our analytical predictions remain true up to times of the order of the inverse standard deviation of the energy. To see this, note the following standard bound on how much $|\psi\rangle$ dynamically deviates from being an eigenstate:

$$f(t) = \left\| \mathrm{e}^{-\mathrm{i}\hat{H}t}|\psi\rangle - \mathrm{e}^{-\mathrm{i}tE}|\psi\rangle \right\|^2 \le 2t\sqrt{\mathrm{Var}(|\psi\rangle, \hat{H})},$$

where $E = \langle\psi|\hat{H}|\psi\rangle$. To derive this bound, we first compute the derivative of the squared norm as

$$\begin{aligned}\frac{\mathrm{d}}{\mathrm{d}t}f(t) &= \frac{\mathrm{d}}{\mathrm{d}t}\left(2 - (\langle\psi|\mathrm{e}^{-\mathrm{i}t(\hat{H}-E)}|\psi\rangle + \text{c.c.})\right) \\ &= \mathrm{i}(\langle\psi|(\hat{H}-E)\mathrm{e}^{-\mathrm{i}t(\hat{H}-E)}|\psi\rangle + \text{c.c.}).\end{aligned}$$

Thus, we find that the derivative is upper-bounded as

$$\begin{aligned}\left|\frac{\mathrm{d}}{\mathrm{d}t}f(t)\right| &\le 2\left|\langle\psi|\mathrm{e}^{-\mathrm{i}t(\hat{H}-E)}(\hat{H}-E)|\psi\rangle\right| \\ &\le 2\,\||\psi\rangle\|\,\left\|(\hat{H}-E)|\psi\rangle\right\| \\ &= 2\sqrt{\mathrm{Var}(|\psi\rangle, \hat{H})}. \qquad (12)\end{aligned}$$

Since $f(0) = 0$, we get

$$\begin{aligned}f(t) &= \left|\int_0^t \frac{\mathrm{d}}{\mathrm{d}t'}f(t')\mathrm{d}t'\right| \le \int_0^t \left|\frac{\mathrm{d}}{\mathrm{d}t'}f(t')\right|\mathrm{d}t' \\ &\le 2t\sqrt{\mathrm{Var}(|\psi\rangle, \hat{H})}. \qquad (13)\end{aligned}$$
